# METTL3/m^6^A/miRNA-873-5p Attenuated Oxidative Stress and Apoptosis in Colistin-Induced Kidney Injury by Modulating Keap1/Nrf2 Pathway

**DOI:** 10.3389/fphar.2019.00517

**Published:** 2019-05-15

**Authors:** Jian Wang, Muhammad Ishfaq, Liang Xu, Chunli Xia, Chunli Chen, Jichang Li

**Affiliations:** ^1^College of Veterinary Medicine, Northeast Agricultural University, Harbin, China; ^2^Heilongjiang Key Laboratory for Animal Disease Control and Pharmaceutical Development, Harbin, China

**Keywords:** colistin, m^6^A modification, oxidative stress, apoptosis, miRNA

## Abstract

Nephrotoxicity of colistin is the major factor limiting its clinical application. However, the exact mechanism of colistin-induced nephrotoxicity is still elusive. N^6^-Methyladenosine (m^6^A) modification has been implicated in many biological processes, however, its role in colistin-induced nephrotoxicity needs to be elucidated. Mouse renal tubular epithelial cells (mRTECs) were treated with 200 μM colistin with or without METTL3 overexpression. Cells injury, m^6^A assay, oxidative stress and apoptosis were examined. Levels of m^6^A are decreased after colistin treatment in mRTECs. METTL3 is the major factor involved in abnormal m^6^A modification. METTL3 overexpression plays a protective role against colistin-induced oxidative stress and apoptosis. Moreover, METTL3 interacts with the microprocessor protein DGCR8 and positively modulates miR-873-5p mature process in an m^6^A-dependent manner. Further experiments show that miR-873-5p could regulate Keap1-Nrf2 pathway against colistin-induced oxidative stress and apoptosis. These studies revealed an important role of METTL3/m^6^A in colistin-induced nephrotoxicity and provide a new insight on m^6^A modification in drug induced toxicity.

## Introduction

Researchers extensively demonstrated the increasing proportion of infections caused by multi-drug resistant and pandrug-resistant gram-negative bacteria and the lack of effective treatment drugs, and colistin has become an important choice for the treatment of such infections (Li et al., 2006; Wadl et al., 2010; Hawkey, 2015; Aliyu et al., 2017; Rodrigo-Troyano and Sibila, 2017). However, due to side effects of colistin and the lack of clinical application experience, there are no appropriate corresponding guidelines for reference all around the world, and clinicians often have misgivings about the use of these drugs (Wertheim et al., 2013). The main adverse reactions of colistin is dose dependent nephrotoxicity and neurotoxicity including allergic reaction (itching, contact dermatitis, drug fever and so on), ototoxicity, liver toxicity, mild gastrointestinal reaction and partial dosing irritation. In addition, it has been reported that colistin can cause cough, bronchial contraction and chest distress after inhalation (Dijkmans et al., 2015; Loho and Dharmayanti, 2015). However, except nephrotoxicity, the incidence of other adverse reactions of colistin is low and symptoms are mild, which can be recovered after drug withdrawal. Neurotoxicity is one of the main adverse reactions of colistin, but in the past few decades, reports of neurotoxicity caused by this drug are rare (Gupta et al., 2009; Weinstein et al., 2009). Therefore, nephrotoxicity is the most common adverse reactions of colistin and it is necessary to clarify the underlying mechanism of colistin-induced renal injury.

Reactive oxygen species (ROS) generated by oxidative stress will directly or indirectly damage the physiological functions of proteins, lipids, nucleic acids and other macromolecules in cells, which is the pathophysiological basis of many diseases. Many reports have indicated that oxidative stress is an important factor in cell apoptosis, which plays an important role in the process of renal toxicity induced by colistin (Dai et al., 2014, 2017; Edrees et al., 2018; Jeong et al., 2018). Based on the molecular mechanism of oxidative stress, antioxidant stress is an important protective measure to inhibit the renal toxicity of colistin.

Keap1-Nrf2/ARE antioxidant pathway plays an important role in cellular antioxidant stress. Nrf2 and Keap1 are central regulators of antioxidant responses in cells, and Nrf2 binds with Keap1 in a relatively inhibitory state. Keap1 separates from Nrf2 and Nrf2 starts moving into the nucleus to induce phase-II detoxifying enzymes and antioxidant enzyme gene expression (Shelton et al., 2013; Dai et al., 2015). Nrf2 deletion or abnormal activation could further aggravate the cytotoxicity of oxidative stress, leading to cell dysfunction, apoptosis and even death (Liu et al., 2009; Ruiz et al., 2013; Shelton et al., 2013; Dai et al., 2015; Chen et al., 2016). Keap1 plays a key regulatory role in the Nrf2 pathway, but the underlying mechanism of its regulation is still unknown.

MicroRNAs (miRNAs) are a class of endogenous non-coding RNA with regulatory functions found in eukaryotes, with a size of about 20–25 nucleotides. Mature miRNAs are produced by the shear processing of a series of nucleases on longer primary transcripts, which are then assembled into RNA-induced silencing complexes to identify target mRNA by complementary base pairing, and guide the silencing complexes to degrade target mRNA or inhibit the translation of target mRNA (Du and Zamore, 2005).

RNA N^6^-Methyladenosine (m^6^A) modification was first reported in 1974, and subsequent studies found that RNA m^6^A modification was widely found in various tissues and organs, and showed dynamic and reversible changes in different tissues at different stages of development (Jia et al., 2011; Zheng et al., 2013; Batista et al., 2014; Liu et al., 2014; Ping et al., 2014; Geula et al., 2015; Ma et al., 2017). RNA m^6^A modification is the most abundant RNA modification in eukaryotes and highly conserved among multiple species. Recent studies have shown that RNA m^6^A modification is catalyzed by the dynamic regulation of methyltransferase and demethylase. Methyltransferase include METTL3, METTL14, WTAP, KIAA1429, etc. (Ma et al., 2017). The demethylases include FTO and ALKBH5 (Ma et al., 2017). As an important epigenetic modification, RNA m^6^A modification is related to cell development, maintenance of stem cell characteristics, mitotic control, circadian rhythm regulation, fertilization ability, and tumorigenesis ability (Bokar et al., 1997; Jia et al., 2011; Zheng et al., 2013; Batista et al., 2014; Liu et al., 2014; Ping et al., 2014; Geula et al., 2015; Ma et al., 2017). However, the role and function of m^6^A modification in drug-induced toxicity remains to be further studied. It has been reported that m^6^A can label pri-miRNAs and identify DGCR8 molecules by METTL3/m^6^A, participating in the mature process of miRNAs and leading to differential expression of miRNAs in many biological processes (Alarcón et al., 2015; Ma et al., 2017). It has been reported that miRNA can target Keap1 to inhibit its expression and thus activate the Nrf2 pathway (Yang et al., 2014; Kabaria et al., 2015; Akdemir et al., 2017). We hypothesized that METTL3 is also involved in the maturation process of miRNAs during the occurrence of colistin-induced nephrotoxicity, resulting in the expression difference of anti-oxidative stress related miRNA. The results showed that miR-873-5p was regulated by METTL3-mediated m^6^A modification, which enhanced DGCR8 recognition of pri-miR-873, promoted the generation of mature miR-873-5p, miR-873-5p targeted inhibition of Keap1, and then activated the Nrf2/HO-1 pathway. The present study describes that METTL3 protect against colistin-induced nephrotoxicity and examine the molecular mechanisms in an *in vitro* model.

## Materials and Methods

### Reagents and Antibodies

Colistin sulfate was obtained from Sigma-Aldrich (catalog number: C4461, St. Louis, MO, United States). Fetal bovine serum (FBS) and Dulbecco’s modified Eagle medium (DMEM) was purchased from Gibco (catalog number: 10099141C and 12491-015, Gaithersburg, MD, United States). Lipofectamine 2000 and annexin V-FITC/PI staining kit was purchased from Invitrogen (catalog number: 11668-027 and V13242, Grand Island, NY, United States). Primary antibodies against METTL3 (1:500 dilution), Nrf2 (1:1,000 dilution) and HO-1 (1:1,000 dilution) were obtained from Bioss (catalog numbers: bs-17609R, bs-1074R, and bs-2075R, Woburn, MA, United States). Anti-β-actin rabbit monoclonal antibody (1:1,000 dilution) and secondary antibodies (1:5,000 dilution) were obtained from Santa Cruz Biotechnology (catalog numbers: sc-376421 and sc-2357, Santa Cruz, CA, United States). Antibodies for DGCR8 (1:500 dilution) and m^6^A (1:500 dilution) and m^6^A RNA methylation quantification kit were from Abcam (catalog numbers: ab90579, ab208577, and ab185912 Cambridge, MA, United States).

### Cell Culture and Treatment

Mouse renal tubular epithelial cells (mRTECs) were obtained from the Cell Bank of the Type Culture Collection, Shanghai Institute of Cell Biology, Chinese Academy of Sciences. The cells were cultured in DMEM supplemented with 10% (vol/vol) FBS at 37°C in 5% CO_2_ atmosphere in a CO_2_ incubator. There are four groups given as follows: control group (serum-free DMEM), colistin treated group (cells incubated with 200 μM colistin for 24 h), METTL3 overexpression negative control group and METTL3 overexpression group [the cells were transfected with pcDNA3.1(+) and pcDNA3.1(+)-METTL3 following the treatment with 200 μM colistin for 24 h, respectively]. MiR-873-5p overexpression negative control group and MiR-873-5p overexpression group (the cells were transfected with negative control mimic and miR-873-5p mimic following the treatment with 200 μM colistin for 24 h, respectively).

### Plasmid Construction and Transfection

The pcDNA3.1(+) plasmid were purchased from synbio-tech Co., Ltd. (Jiangsu, China). The pcDNA3.1(+)-METTL3 plasmid was cloned via the insertion of the METTL3 gene sequence (GenBank Accession No: NM_019721.2) into pcDNA3.1(+). Construction process as follows: total mRNA from mRTECs was used as a template for cDNA and was used to amplify the METTL3 coding sequence. The METTL3 specific primers as follows: Forward: 5′-TAGCATCTGGTCTGGCCTCT-3’; Reverse: 5’-GGCCCTGGTTGAATCCTTGA-3’; The amplified METTL3-coding sequence was digested with *Bam*HI and *Xho*I, and then ligated into *Bam*HI and *Xho*I digested pcDNA3.1(+). The mRTECs were seeded at a density of 5 × 10^5^ cells per well for 12 h at 37°C in a humidified CO_2_ incubator. At the time of the confluence reached 80%, Lipofectamine 2000^TM^ was used for transfection according to the manufacturer’s instructions. For each transfection, 2.5 μg of pcDNA3.1(+)-METTL3/pcDNA3.1(+) was prepared and overlaid onto the cells separately in DMEM medium and incubated for 8 h. Then, the medium was removed and replaced with 10% FBS/DMEM for 24 h following the treatment with 200 μM colistin for 24 h, the METTL3 overexpression efficiency was assessed by western blot. The pcDNA3.1(+) were used as negative controls. The miR-873-5p mimic and negative control mimic were obtained from GenePharma Co., Ltd. (Shanghai, China). A concentration of 62.5 nmol/L miR-873-5p mimic and negative control mimic were transfected into cells for 48 h with Lipofectamine 2000^TM^ (Invitrogen) according to manufacturer’s protocol. Then, the medium was removed and replaced with 10% FBS/DMEM for 12 h following the treatment with 200 μM colistin for 24 h. Sequence of miR-873-5p mimics included: Sense: 5′-GCAGGAACUUGUGAGUCUCCU-3′; Antisense: 5′-GAGACUCACA AGUUCCUGCUU-3′. Negative control mimics included: Sense: 5′-UUGACGCU UCGUGUCACGUTT-3′; Antisense: 5′-ACGUGACACGAAGCGUCAAT-3′.

### Luciferase Reporter Assay

The fragment of wild-type Keap1 including binding sites for miR-873-5p was amplified by PCR using primers with restriction endonuclease sites, Forward: GGAAGATCTTTTACCAGGAAATGACCAAATCTGAG; Reverse: CCGGGATC CTTAATTTAATTTGGCAGTCATAATAAT. The mutant Keap1 fragment was obtained by overlap PCR (AAATTATTGCAAAACTCCAAACTTTAATTATTCGA AACTCCAACAGA). The fragment of wild-type Keap1 and mutant Keap1 were inserted into *Bgl*II/*Bam*HI digested pGL3 vector with a firefly and luciferase reporter gene. The fragment of miR-873-5p was amplified by using primers with restriction endonuclease sites (Forward: CCGCTCGAGGCTCTTCAGCATGTC ACATGCC; Reverse: CCGGGATCCCGCATTCGACCTAACCAACACC) and were ligated into *Bam*HI/*Xho*I digested pGMLV vector. For transfection, human 293T cells were plated in DMEM medium (Gibco) in a 24-well plate. After 24 h, the cells were transfected with 200 ng pGMLV vector with mmu-miR-873-5p fragment or negative control with the pGL3 vectors with wild-type Keap1 or mutant Keap1 fragments, respectively, using Lipofectamine^TM^ 2000 (Invitrogen) according to the manufacturer’s instructions. The luciferase activities were detected by using a dual-luciferase reporter assay system (catalog number: E1910, Promega) following the manufacturer’s protocol.

### MTT and LDH Assay

Briefly, mRTECs were seeded at 1 × 10^5^ cells/well in 96-well plates and incubated for 12 h. After the cells were treated with a series dose of colistin for 2–48 h, the medium was discarded. 20 μL MTT solution (5 mg/mL, catalog number: M2128, Sigma-Aldrich) was added and the plates were incubated at 37°C for 4 h. Then, the medium was removed and 100 μL DMSO (catalog number: D2650, Sigma-Aldrich) was added to each well and incubated for 20 min at room temperature. Finally, the absorbance of each well was measured at 490 nm by a microplate reader (Bio-Rad, Hercules, CA, United States). LDH assay kit purchased from Nanjing Jiancheng Bio-Corporation (catalog number: A020-2-2, Jiangsu, China). LDH content was measured according to the manufacturer’s instructions. Briefly, after colistin treatment, the medium was collected and centrifuged at 3,500 rpm for 10 min at 4°C. The supernatant was collected and then the absorbance was measured with a microplate reader (Bio-Rad, Hercules, CA, United States) at 490 nm.

### Measurement of SOD, GSH-PX, MDA, and CAT

The activities of SOD (catalog number: A001-1-2) and CAT (catalog number: A007-1-1), and the contents of GSH-PX (catalog number: A005-1-2) and MDA (catalog number: A003-1-2) in cells from each group were measured by using commercial assay kits (Nanjing Jiancheng Bio-Corporation). All group cells were washed with PBS and lysed using the cell lysis buffer provided by the manufacturer. The cell lysates were centrifuged at 12,000 rpm for 15 min. Supernatants were collected, after incubation with a series of reagents, the content or activity were measured by using a multimode plate reader (Thermo Fisher Scientific, United States).

### ROS Measurement

Cells were collected from each group. After three washes with cold PBS, the DCFH-DA fluorescence was imaged using a fluorescent microscope at excitation wavelength 488 nm, emission wavelength 530 nm (Olympus, Japan). ROS content was measured according to the manufacturer’s instructions (catalog number: E004-1-1 Nanjing Jiancheng Bio-Corporation, Jiangsu, China). Cell lysates were collected by 3,500 rpm for 10 min at 4°C. After incubation with a series of reagents, the fluorescence was measured by using a multimode plate reader (Thermo Fisher Scientific, United States).

### Measurement of m^6^A Modification

Total RNA from each group was extracted using TRIzol (catalog number: ET101-01, TransGen, Beijing, China) according to the manufacturer’s protocol. The quality of RNA was measured by NanoDrop (Thermo Fisher Scientific, United States). The m^6^A content measured by using a commercial kit (catalog number: ab185912, Cambridge, MA, United States). Briefly, 200 ng RNAs were plated on assay dishes. Capture antibody solution and detection antibody solution were then added. The m^6^A levels were quantified by reading the absorbance of each sample at a wavelength of 450 nm by a microplate reader (Bio-Rad, Hercules, CA, United States).

### RNA Isolation and Quantitative Real-Time Polymerase Chain Reaction (qRT-PCR)

Total RNA was isolated from cells using TRIzol reagent following the manufacturer’s instructions. Three micrograms of total RNA were reverse transcribed using the Transcriptor First Strand cDNA Synthesis Kit (catalog number: AT301-02, TransGen, Beijing, China). The levels of mRNA were determined using real-time PCR with a SYBR premix Ex Taq kit (catalog number: AQ101-03, TransGen, Beijing, China) on an Applied Biosystems 7500 real-time PCR system thermocycler. Each sample was analyzed in triplicate. The β-actin gene was used as a control for the calculation of ΔCt. The data were analyzed through 2^-ΔΔCt^ method. Forward (F) and reverse (R) primers were as follows: Nrf2-F: 5′-CCCCTGGAAGTGTCAAACAG-3′, Nrf2-R: 5′-CACATTG GGATTCACGCATA-3′; HO-1-F:5′-TTG ACTGACCACGACTGCTG-3′, HO-1-R: 5′-ACAAGACAGAAATACGAGACAGA-3′; Keap1-F: 5′-GGCAAGATCTACGT CCTCGG-3′, Keap1-R: 5′-ACCGAGTTGCAGGAACCAAA-3′; For detection of mature miR-873-5p, cDNA was synthesized with the miRNA First Strand cDNA Synthesis (catalog number: B532453-0020, Shanghai sangon, Shanghai, China) and U6 snRNA was used as a control. The loop-RT primers for miR-873-5p were as follows: GTCGTATCCAGTGCAGGGTCCGAGGTATTCGCACTGGATACGACCT ACCA. The loop-RT primers for U6 were as follows: CGAGCACAGAATCGCTTCA CGAATTTGCGTGTCAT. MicroRNAs Quantitation PCR Kit (catalog number: B532461-0002, Shanghai sangon, Shanghai, China) used to measure miR-873-5p expression. Primer sequences were as follows: miR-873-5p-F: 5′-CGCATGGCA GTGGTTTTACCCTA-3; miR-873-5p-R: 5′-ATCCAGTGCAGGGTCCGAGG-3′; U6-F: 5′-CGCTTCGGCAGCACATATACTAAAATTGGAAC-3′; U6-R:5′-GCTTC ACGAATTTGCGTGTCATCCTTGC-3′; Pri-miR-873-5p-F: 5′-GCCATTTCAGTG GGCTTACAATA-3′; Pri-miR-873-5p-R: 5′-CGTTGUTGCAGGGGAAGAGG-3′. Fold changes in expression of each gene were calculated by a comparative threshold cycle (Ct) method using the formula 2^-ΔΔCt^.

### RNA Immunoprecipitation (RIP)

RIP experiments were performed using the Magna RIP Kit (catalog number: Millipore-MAGNARIP03, Millipore, MA, United States) as described previously (Ma et al., 2017). Briefly, cells from each group were UV-irradiated and lysed at 48°C through disruptive sonication. Immunoprecipitations of DGCR8 were performed using an anti-DGCR8 antibody overnight at 48°C. After two times washing, RNAs was extracted and subjected to qRT-PCR using primers for primary microRNAs (pri-miRNAs) and normalized to input. The m^6^A RNA binding experiments was similar to the above process. Cells were isolated and treated with deoxyribonuclease I, and then fragmented by sonication for 8–15 s. Immunoprecipitations were carried out using an anti-m^6^A antibody and incubated with fragmented RNAs, then treated with proteinase K for 1–2 h at 48°C. RNAs was extracted and subjected to qRT-PCR using primers for pri-miRNAs and finally normalized to input.

### Co-immunoprecipitation

Co-immunoprecipitation was performed using the Pierce^TM^ co-immunoprecipitation Kit (catalog number: 26149, Thermo Fisher Scientific, United States). Anti-METTL3, Anti-DGCR8 and the negative control IgG antibody were used, the immune compounds were treated RNase for 5 min for 37°C, then carries on the Western Blot.

### Measurement of Caspase-3 and Caspase-9 Activity

Caspase-3/caspase-9 enzyme activity was measured according to the instructions of the detection kits (catalog numbers: G015-1-3 and G018-1-1, Nanjing Jiancheng Bio-Corporation, Jiangsu, China). Absorbance was measured at 405 nm based on caspase-3/caspase-9 and can catalyze the substrate ac-devd-pna/ac-lehd-pna to produce yellow pNA with strong light absorption value at 405 nm. In brief, 10^4^ cells were cultured and collected from each group followed by adding the reaction solution contained in the kit, and OD value was measured at 405 nm with a multimode plate reader (Thermo Fisher Scientific, United States).

### Western Blot

Western blot procedure was carried out as mentioned in earlier study (Dai et al., 2014). Briefly, the extracted protein were separated by 10 or 12% SDS-PAGE, shifted to a polyvinylidene difluoride membrane (catalog number: abs931, Absin, Shanghai, China), which were incubated with primary antibodies against METTL3, DGCR8, Keap1, Nrf2, and HO-1. The membranes were then washed in TBST (catalog number: abs952, Absin, Shanghai, China) and incubated with secondary antibodies correspondingly. After TBST washing, bands were visualized with ECL kit (catalog number: abs920, Absin, Shanghai, China). All western blot images showed in Supplementary Material section.

### DNA Fragmentation and Cyt-c Detection

DNA fragmentation and Cyt-c detection was carried out according to the instructions of Cell Death Detection ELISA PLUS Detection kit (catalog number: H190, Nanjing Jiancheng Bio-Corporation, Jiangsu, China). 10^4^ cells were collected from each group, and the reaction solution contained in the kit was added in turn. OD value was measured at 405 nm with a microplate analyzer (Thermo Fisher Scientific, United States). The degree of DNA fragmentation was expressed as a percentage of the control group.

### Apoptosis Assay

The apoptosis was measured by using an Annexin V-FITC apoptosis detection kit following the manufacturer’s instruction (catalog number: abs920, Absin, Shanghai, China). Cells were collected and washed twice by cold PBS and resuspended in 300 μL binding buffer supplied by the manufacturer for flow cytometry. The cells were incubated with 5 μL annexin V-FITC (40 μg/mL) in the dark for 15 min. Five microliters of propidium iodide (PI) (40 μg/mL) was added before testing and finally added 200 uL binding buffer. Apoptotic cells were quantitated by flow cytometry (Coulter Epics XL, Beckman Coulter, United States).

### Statistical Analysis

Data (obtained from at least three independent experiments and are presented as means ± SD) from all experimental groups were analyzed with one-way analysis of variance (ANOVA), followed by the LSD *post hoc* test using SPSS v. 21.0 (SPSS Inc., Chicago, IL, United States). A value of *p* < 0.05 was considered as statistically significant.

## Results

### Colistin-Induced Nephrotoxicity *in vitro*

**FIGURE 1:**
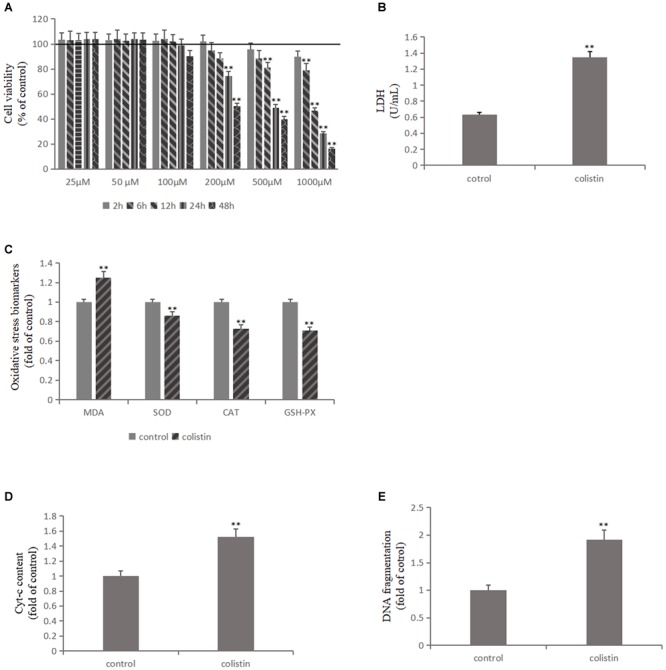
Colistin-induced nephrotoxicity *in vitro*. **(A)** Assessment of cell viability of mRTECs following a series dose of colistin treatment at different time point by MTT assay. **(B)** LDH assay. **(C)** Oxidative stress markers detection. **(D)** Cyt-c content and **(E)** DNA fragment measured by ELISA Kit. All the data were represented by the mean ± SD from at least three independent experiments. Values that are significantly different from the values for the control group are indicated by asterisks as follows: ^∗∗^*p* < 0.01.

The effects of 25, 50, and 100 μM colistin showed a certain growth-promoting effect (Figure 1A). The cell survival rate decreased with the increase of 200, 500, and 1000 μM (Figure 1A). The cell survival rate decreased significantly (*p* < 0.01) with prolong exposure time of colistin (Figure 1A). It has been noted that when the concentration of colistin increased to 200 μM for 24 h, obvious cell damage is seen, and the cell vitality was significantly (*p* < 0.01) decreased relative to control group (Figure 1A). Therefore, an *in vitro* model of renal toxicity was established by using 200 μM colistin-treated cells for 24 h.

Lactate dehydrogenase (LDH) exists in cells of almost all tissues and its concentration increased upon cell damage. Therefore, the degree of cell damage can be determined by detecting the activity of LDH in the culture medium. After colistin treatment for 24 h, the LDH content in cell culture medium was significantly increased (*p* < 0.01, Figure 1B), indicating severe cell damage. We further measured the ROS content, lipid peroxidation marker MDA and the activity of antioxidant enzymes SOD, CAT, and GSH-PX in cells, and the results confirmed that the ROS and MDA levels were significantly increased and the activity of SOD, CAT, and GSH-PX was significantly (all *p* < 0.01) decreased after 24 h of 200 μM colistin treatments (Figure 1C). These changes suggest that oxidative stress was involved in colistin-induced renal injury. Subsequently, the level of apoptosis-related indicators was further detected. Compared with the control group, the activity of Caspase-3, Caspase-9, the level of Cyt-c and DNA fragment were significantly (all *p* < 0.01) increased after 24 h of treatment with 200 μM colistin (Figure 1D,E, 3A). The apoptosis rate was further analyzed by flow cytometry. The results showed that apoptosis rate was also significantly (*p* < 0.01) increased (Figure 3B). The above results further confirmed the reliability of the *in vitro* model of colistin-induced nephrotoxicity.

### METTL3 Is Responsible for the Abnormal m^6^A Modification

**FIGURE 2:**
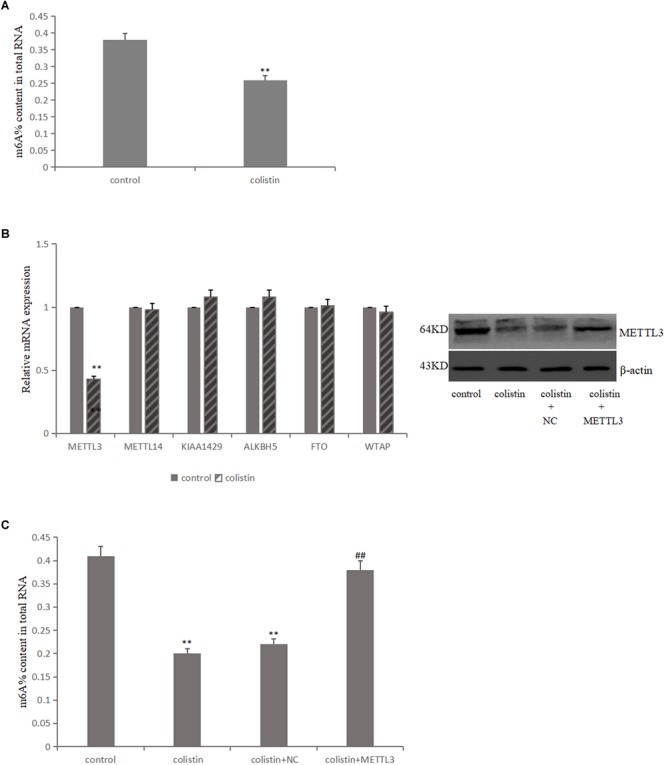
Aberrant m^6^A modification involved in colistin-induced renal injury. **(A)** Effect of colistin on m^6^A contents of total RNAs in mTRECs. **(B)** mRNA levels of m^6^A modification-associated genes and immunoblotting of METTL3 after colistin treatment. **(C)** Effect of METTL3 overexpression on m^6^A contents of total RNAs. Values that are significantly different from the values for the control group are indicated by asterisks as follows: ^∗∗^*p* < 0.01. Values that are significantly different from the values for the colistin group are indicated as follows: ^##^*p* < 0.01.

In order to study the effect of m^6^A modification on the nephrotoxicity of colistin, m^6^A levels were detected in mRTECs under different experimental treatments The results showed that m^6^A levels significantly (*p* < 0.01) decreased after 200 μM colistin treatment for 24 h (Figure 2A). This suggests that reduced m^6^A levels may be involved in colistin-induced nephrotoxicity.

The m^6^A modification is mainly catalyzed and regulated by m^6^A methyltransferase and demethylase, it is then speculated that whether the decreased m^6^A levels was caused by the differential expression of key m^6^A methylase and demethylase. To test the above hypothesis, the key gene expression level of m^6^A was detected. The results showed that the expression of METTL3 was significantly down-regulated after colistin treatment for 24 h compared to control group. While the expression levels of METTL14, FTO, ALKBH5, WTAP and KIAA1429 genes were not significantly different (Figure 2B). Further, METTL3 was overexpressed in mRTECs and the results showed that the decrease of METTL3 expression was the main reason for the decrease of m^6^A levels caused by colistin (Figure 2C).

### METTL3 Plays a Protective Role in Colistin-Induced Renal Injury

**FIGURE 3:**
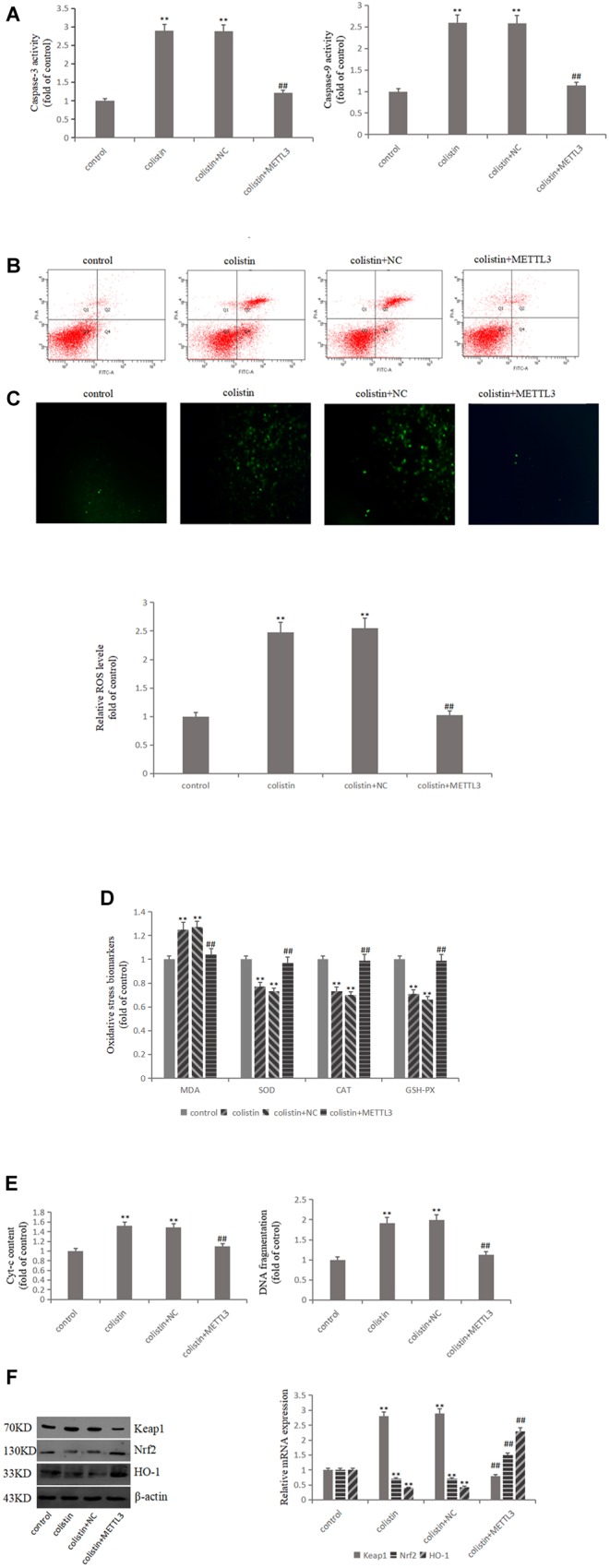
METTL3 overexpression reduces oxidative stress and apoptosis after colistin treatment. **(A)** Activity of Caspase-3 and Caspase-9 in each group. **(B)** Apoptosis of mRTECs was analyzed by flow cytometry following annexin V-FITV/PI staining. Q1, necrosis cells; Q2, later apoptotic cells; Q3, live cells; Q4, early apoptotic cells. **(C)** METTL3 overexpression attenuates colistin-induced ROS production. **(D)** Oxidative stress markers in each group. **(E)** Cyt-c content and DNA fragment measurement. **(F)** Western blot and qPCR analysis of Keap1, Nrf2, and HO-1 expression. The values that are significantly different from the values relative to control group are indicated by asterisks as follows: ^∗∗^*p* < 0.01. Values that are significantly different from the values of colistin-treated group are indicated as follows: ^##^*p* < 0.01.

In order to study the relationship between METTL3 and colistin-induced nephrotoxicity, we overexpressed METTL3 in mRTECs. The results showed that ROS and MDA levels were significantly decreased (all *p* < 0.01), and the activities of SOD, CAT, and GSH-PX were significantly (all *p* < 0.01) increased in overexpressed METTL3 group compared to colistin treatment (Figure 3C,D). These results suggest that METTL3 may alleviate colistin-induced renal injury by an anti-oxidative stress manner. The overexpressed METTL3 group results also showed that DNA fragment content and Cyt-c release, the activity of Caspase-3 and Caspase-9 were significantly (all *p* < 0.01) lower than those in the colistin treated group (Figure 3A,E), overexpressed METTL3 significantly (*p* < 0.01) reduced apoptosis rate of cells treated with 200 μM colistin for 24 h (Figure 3B). These results suggest that METTL3 may alleviate colistin-induced renal injury by an anti-apoptosis manner. In addition, overexpression of METTL3 can reduce the increase of Keap1 expression caused by colistin treatment, and significantly (*p* < 0.01) enhance colistin-induced decrease of Nrf2 and HO-1 (Figure 3F), suggesting that the anti-oxidative stress and anti-apoptotic effect of METTL3 may involve Keap1/Nrf2 pathway.

### METTL3-Dependent m^6^A Methylation Regulates Mature Process of miR-873-5p in Colistin-Induced Renal Injury

**FIGURE 4:**
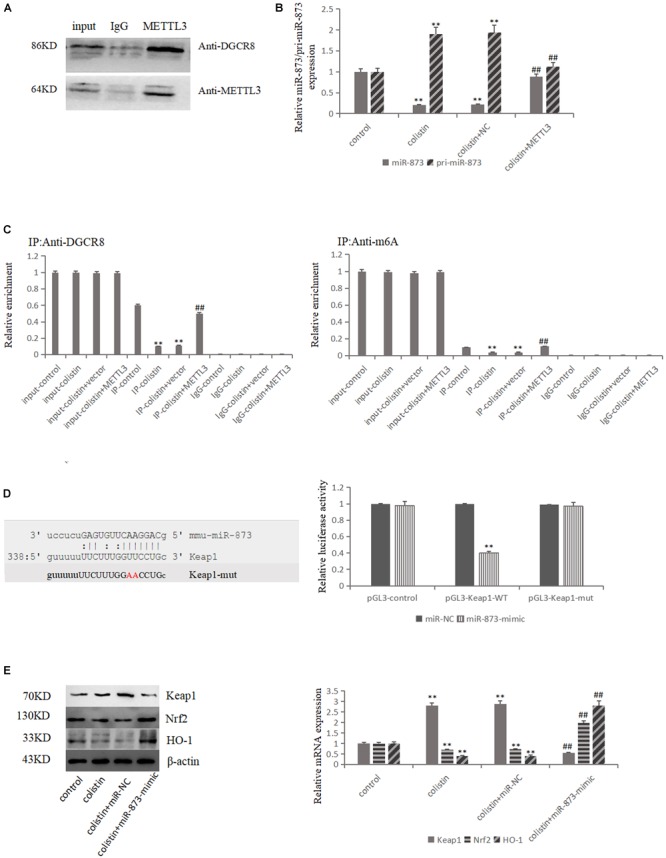
METTL3-dependent m^6^A methylation regulates the processing of mmu-miR-873-5p by DGCR8. **(A)** Co-IP of the METTL3-interacting protein DGCR8. mREECs were crosslinked before the immunoprecipitation. IgG antibody was used as control. **(B)** MiR-126 and pri-miR126 were quantified by qPCR in all groups. **(C)** IP of DGCR8-associated RNA from control or METTL3-overexpressing cells followed by qPCR to detect pri-mmu-miR-873-5p binding toDGCR8. Immunoprecipitation of m^6^A modified RNA in control or METTL3-overexpressing cells followed by qPCR to assess the pri-mmu-miR-873-5p m^6^A modification levels. **(D)** Bioinformatics analysis of predicted interactions of mmu-miR-873-5p with its binding sites in Keap1. Luciferase activity was measured by dual-luciferase reporter assay. Luciferase activity was normalized to Renilla luciferase activity. **(E)** Western blot and qPCR analysis of Keap1, Nrf2, and HO-1 expression. The values that are significantly different from the values relative to control group are indicated by asterisks as follows: ^∗∗^*p* < 0.01. Values that are significantly different from the values of colistin-treated group are indicated as follows: ^##^*p* < 0.01.

DGCR8, the major partner of type III RNase Drosha, plays an important role in miRNA biogenesis generally via interacting with pri-miRNA and recruiting Drosha to cleave pri-miRNA at the correct site into pre-miRNA. Then, the pre-miRNA is cleaved by Dicer RNase to mature miRNA. In order to detect whether METTL3 is involved in the miRNA maturation process, we conducted co-IP experiments with METTL3 antibody in mRTECs. The results showed that METTL3 could bind DGCR8 (Figure 4A), suggesting that METTL3 may participate in the miRNA maturation and play a role in regulating DGCR8 recognition and binding pri-miRNAs.

Two bioinformatics software (miRDB and microrna.org) were used to predict miR-873-5p that could target Keap1, and the luciferase reporter vector experiment confirmed the targeting relationship (Figure 4D). The expression levels of miR-873-5p and pri-miR-873 were detected in each group. The results showed that miR-873-5p significantly (*p* < 0.01) decreased after colistin treatment in comparison with control group. Compared with the colistin treatment group, METTL3 overexpression could significantly (*p* < 0.01) increase miR-873-5p expression. The expression trend of pri-miR-873-5p was opposite to that of miR-873-5p (Figure 4B). Furthermore, the pri-miR-873-5p expression level was detected in each group after RIP with DGCR8 antibody. The results showed that the level of pri-miR-873 bound with DGCR8 was significantly (*p* < 0.01) decreased after colistin treatment. METTL3 overexpression could increase the level of pri-miR-873 bound with DGCR8 (Figure 4C), suggesting that the overexpression of METTL3 could promote DGCR8 recognizing and binding to pri-miR-873. Subsequently, m^6^A RNA binding experiments was performed, and the results revealed that the pri-miR-873 modified by m^6^A was significantly (*p* < 0.01) decreased after colistin-treatment. While, the overexpression of METTL3 could reverse the decreased m^6^A levels of pri-miR-873 caused by colistin treatment (Figure 4C), suggesting that the m^6^A could enhance the recognition of pri-miR-873 by DGCR8 and promote the production of mature miR-873-5p.

**FIGURE 5:**
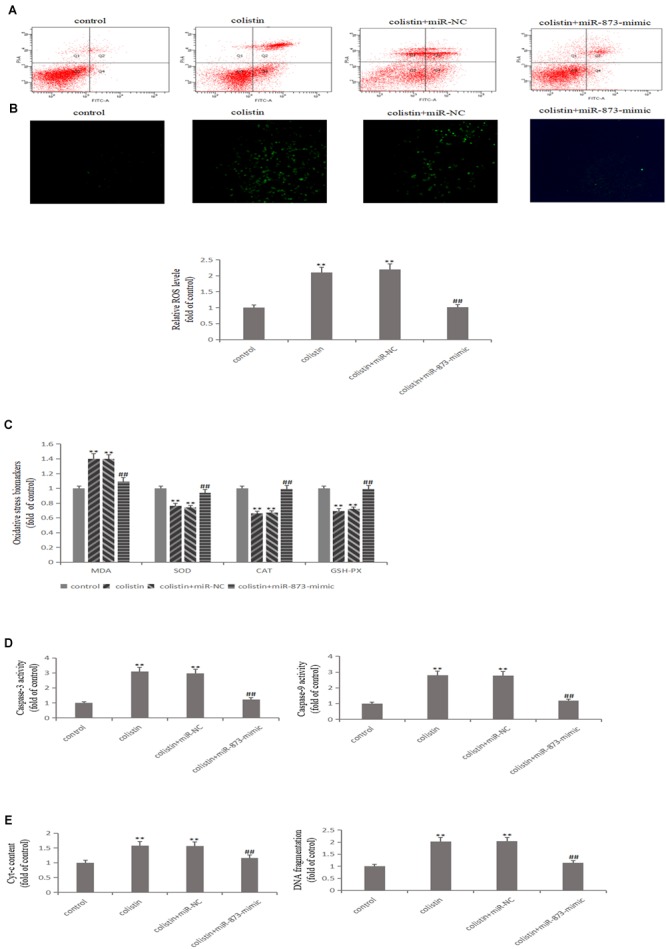
Overexpressed miR-873-5p suppresses oxidative stress and apoptosis. **(A)** Apoptosis of mRTECs was analyzed by flow cytometry following annexin V-FITV/PI staining. Q1, necrosis cells; Q2, later apoptotic cells; Q3, live cells; Q4, early apoptotic cells. **(B)** MiR-873-5p overexpression attenuates colistin-induced ROS production. **(C)** Oxidative stress markers in each group. **(D)** Activity of Caspase-3 and Caspase-9 in each group. **(E)** Cyt-c content and DNA fragment measurement. The values that are significantly different from the values for the control group are indicated by asterisks as follows: ^∗∗^*p* < 0.01. Values that are significantly different from the values of colistin-treated group are indicated as follows: ^##^*p* < 0.01.

After transfection with miR-873-5p-mimic in mRTECs, the effects of miR-873-5p in the nephrotoxicity of colistin were further studied. Compared with the colistin-treated group, miR-873-5p-mimic decreased the Keap1 expression and increased Nrf2 and HO-1 expression after treatment with colistin (*p* < 0.01, Figure 4E). The apoptosis rate was significantly (*p* < 0.01) decreased (Figure 5A), the content of ROS and MDA, DNA fragments and the release of Cyt-c were significantly (all *p* < 0.01) reduced (Figure 5B,C,E). The activity of SOD, CAT and GSH-PX in the miR-873-5p-mimic group was significantly (all *p* < 0.01) increased (Figure 5C). Meanwhile, the activity of Caspase-3 and Caspase-9 were also significantly (*p* < 0.01) decreased (Figure 5D).

## Discussion

In the past 20 years, multi-drug resistant gram-negative bacteria have been spreading all over the world, posing a serious threat to human health (Li et al., 2006; Hawkey, 2015; Aliyu et al., 2017; Rodrigo-Troyano and Sibila, 2017). In addition, due to the lack of new and more effective antibiotics, polymyxins (including polymyxins B and colistin) have been re-emphasized and re-applied in clinical practice, and become the best choice or even the last line of defense for the treatment of multi-drug resistant gram-negative bacterial infections (Dijkmans et al., 2015). Nephrotoxicity is one of the main clinical side effects of polymyxin, with an incidence of up to 60% (Dai et al., 2014, 2017). There is increasing evidence that the current recommended dose is subtherapeutic, increasing the risk for clinical polymyxin-resistance, and a simple increase in dose is bound to increase its renal toxicity. Nephrotoxicity has become the most important limiting factor affecting the clinical application of polymyxins. It is of great significance to study the molecular mechanism for reducing renal toxicity and improving therapeutic index.

Animal experiments showed that colistin was mainly distributed in the renal cortex and proximal renal tubules, and the proximal tubule injury was more serious than the distal tubule (Suzuki et al., 2013; Yun et al., 2015b). Further studies showed that the colistin protype was mainly distributed in mitochondria, endoplasmic reticulum and golgi apparatus of renal tubular epithelial cells (Yun et al., 2015a). In this study, we first constructed an *in vitro* model of colistin nephrotoxicity using mRTECs. The experimental results showed that by addition of 25, 50, and 100 μM colistin in cell showed certain growth promoting effect, while other concentrations of colistin have different degrees of damage, such as cell viability and the survival rate was significantly decreased. In addition, cell survival rate also significantly reduced with increase in dose and longer exposure of colistin. In this experiment, 200 μM colistin treated cells for 24 h were used as the damage dose and time of the *in vitro* model, so as to reflect the physiological status of the body in a more real and objective way, making the *in vitro* model more reliable and stable. Moreover, this cytotoxicity is neither as mild as 100 μM, because the concentration is too low for cell injury nor as severe as 500 and 1000 μM, which is not conducive to the subsequent experiments. Yousef et al. (2012) reported the *in vitro* model of colistin-induced nephrotoxicity that 100 μM colistin-treatment for 24 h caused significant apoptosis in NRK-52E cells; Jeong found that 50 μg/mL (≈45 μM) colistin significantly increased the expression of Nox4 with increased reactive oxygen species production and apoptosis rate in HK-2 cells (Jeong et al., 2018). In the present study, 200 μM colistin significantly increased the production of ROS and MDA, decreased the activity of SOD, GSH-PX, and CAT, and induced apoptosis after 24 h, which further confirmed the reliability of the model. It was further confirmed that oxidative stress and apoptosis were important mechanisms of renal toxicity of colistin. Our previous study found that colistin caused an increase in LDH in the cell supernatant (Liu et al., 2013; Jiang and Li, 2014; Lu et al., 2017). Similarly, LDH content in the supernatant of mRTECs was significantly increased after treatment with 200 μM colistin for 24 h in the present study, which indicated that the cell membrane was seriously damaged. Colome et al. (1993) designed an experiment to investigate the effects of gentamicin, kanamycin, streptomycin, and colistin on phospholipid bimolecular membranes or lipid monolayers *in vitro*, and found that only colistin could destroy the lipid membrane structure. Therefore, in addition to oxidative stress and apoptosis, which are the focus of this study, there may be other mechanisms of renal toxicity of myxomycin, which need to be further studied.

**FIGURE 6:**
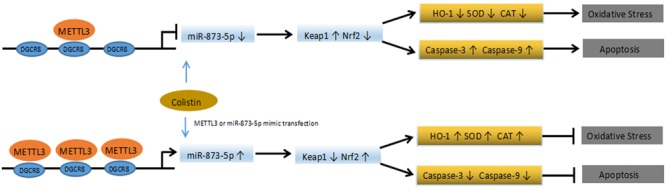
Schematic diagram of the proposed mechanisms of colistin-induced oxidative stress and apoptosis in the present study.

N^6^-Methyladenosine is the most important modification of eukaryotic mRNA. With the advancement in RNA methylation modification study, and how this modification affects normal physiological functions, which leads to diseases has become a hot topic of researchers’ attention. The intracellular dynamic balance of m^6^A is regulated by methyltransferase and demethylase, and the disorder of m^6^A balance have been proved to be closely related to the pathological processes of various tumors (such as glioma, leukemia, breast cancer, hepatocellular carcinoma), obesity, Alzheimer’s disease and HIV infection (Giudice et al., 2010; Vu et al., 2017; Wang et al., 2018). We first examined whether there was a change in m^6^A modification during the nephrotoxicity of colistin. Since there is no simple method for the detection and quantification of m^6^A, m^6^A quantitative kit was used to detect the content of RNA m^6^A in cells. This kit is an ELISA detection kit based on m^6^A antibody specific affinity, and such detection methods have been reported (Ma et al., 2017). The results showed that m^6^A showed a downregulation trend after treatment with colistin. we detected the expression levels of key m^6^A methyltransferase and demethylase involved in the regulation process of m^6^A modification, we found that m^6^A methyltransferase METTL3 was down-regulated in colistin treatment cells. In the present study, METTL3 overexpression in mRTECs can restore the down-regulation trend of m^6^A caused by colistin treatment. Therefore, we believe that METTL3 is a key regulatory gene that leads to the down-regulation of m^6^A. Previous reports have showed that METTL3 is involved in a variety of biological processes. Wang found that METTL3-mediated m^6^A modification is required for cerebellar development (Wang et al., 2018). Song found that METTL3 regulate m^6^A modification of TFEB mRNA, which dictates the fate of hypoxia-treated cardiomyocytes (Song et al., 2019). Xu found that METTL3-mediated m^6^A regulates spermatogonial differentiation and meiosis initiation (Xu et al., 2017). Subsequently, we further studied the biological function of METTL3. Overexpression of METTL3 *in vitro* can alleviate the renal toxicity caused by colistin. Specifically, it can reduce apoptosis, enhance the ability of anti-oxidative stress, reduce the production of ROS, significantly up-regulate Nrf2 and HO-1, and down-regulate Keap1. Nrf2 is a kind of important transcription factors, belongs to CNC leucine zipper, activation of transcription factor family Nrf2-Keap1-ARE mediated phase-II detoxifying enzymes and antioxidant gene transcription was considered the most important cellular antioxidant pathways (Ruiz et al., 2013; Shelton et al., 2013). Under the stimulation of exogenous factors (toxins, inflammation, and ROS), Nrf2 signaling pathway usually has two response modes: early response increases the phosphorylation level at GSK3 Ser9 by activating PKC signaling pathway, promotes the transfer of Nrf2 from cytoplasm to nucleus, and induces the expression of related protective genes in cells; the late response activates GSK3 by phosphorylation at Tyr216, induces Nrf2 to leave the nucleus and bind to Keap1 for degradation, leading to decreased cell stress capacity (Giudice et al., 2010). Mossine found that colistin reduced the activity of the stress-responsive transcriptional factor Nrf2 (Mossine et al., 2018). In addition, Lee found that colistin inhibited the expression of the target genes of Nrf2, which are associated with proteostasis (Lee et al., 2019) and Dai found lycopene and baicalein attenuates colistin-induced nephrotoxicity in mice via activation of the Nrf2/HO-1 pathway (Dai et al., 2015, 2017). Similar to previous results, Nrf2, regulated by METTL3, also plays an important protective role in this experiment. Compared with the colistin-treated group, METTL3 overexpression significantly reduced Keap1 expression, and increased Nrf2 and HO-1 expression. Meanwhile, Apoptosis rate, MDA and ROS were significantly reduced, the activity of antioxidant SOD, CAT, and GSH-PX was significantly elevated, suggesting that METTL3 increased the anti-stress ability of cells. DGCR8 is an important binding protein of type III RNase Drosha, through its two double-stranded RNA binding regions to bind pri-miRNA, and then recruits Drosha to perform shear processing of pri-miRNAs (Denli et al., 2004; Gregory et al., 2004; Alarcón et al., 2015). It has been reported that m^6^A modification can label pri-miRNAs and identify DGCR8 by METTL3/m^6^A, thus participating in the maturation of miRNAs and leading to differential expression of miRNAs in many biological processes (Alarcón et al., 2015; Ma et al., 2017). miRNAs play an important role in kidney damage during exogenous chemical injury. It has been reported that the use of erythropoietin treatment in rats caused an increase in Wnt and catenin expression. While, miR-21, miR-214, miR-210, and miR-199a showed downregulation, which promoted renal tubular epithelial cell proliferation and inhibition of renal tubular epithelial cell apoptosis to protect kidney damage (Chen et al., 2015). Previous study also found that xenon pretreatment of Wistar rats could increase the expression of miR-21 and thus antagonistic to gentamicin-induced nephrotoxicity in the study (Jia et al., 2013). Based on the above understanding, we hypothesized whether METTL3 is also involved in the maturation process of miRNAs during nephrotoxicity of colistin, resulting in the differential expression of miRNA. Through co-IP, RIP and other experiments, we found that miR-873-5p is regulated by METTL3-mediated m^6^A modification, which can enhance DGCR8 recognition of pri-miR-873 and promote the generation of mature miR-873-5p, miR-873-5p plays an anti-oxidative stress and anti-apoptotic role in an *in vitro* model of colistin-induced nephrotoxicity via regulating Keap1/Nrf2 pathways (Figure 6). Our findings reveal the protective role of METTL3/m^6^A/miR-873-5p in colistin-induced nephrotoxicity. However, we cannot exclude the role of other differentially expressed miRNA recognized by METTL3-DGCR8 through dependent m^6^A mechanisms in colistin-induced nephrotoxicity. Alarcón found that METTL3 depletion reduced the binding of DGCR8 to pri-miRNAs and resulted in the global reduction of mature miRNAs, which revealed that m^6^A modification was positively correlated with many miRNAs in a non-cell type-specific manner (Alarcón et al., 2015). Gu et al. (2018) identified nine miRNAs mediated by m^6^A in arsenite-transformed cells. KEGG pathway analysis results revealed that these nine miRNAs were involved in apoptosis, p53 signaling pathway, mTOR signaling pathway and MAPK signaling pathway. Here, we merely identified miR-873-5p as a downstream target of METTL3. Further studies are needed to identify the other target miRNAs of METTL3. Although mice and humans are similar in terms of genomic and physiological characteristics and have achieved a large number of results related to basic biology, there are still limitations in revealing human biology. The results of this experiment are derived from mRTECs, which provide some insights into the mechanism of colistin-induced nephrotoxicity, but further *in vivo* experiments and the application of translational medicines need to be studied in depth.

## Conclusion

In conclusion, we found that m^6^A levels presented a downregulation trend in the *in vitro* model of colistin-induced nephrotoxicity. METTL3 is the main reason for the down-regulation of m^6^A modification. The METTL3-dependent m^6^A modification can regulate the maturation process of miR-873-5p through DGCR8, thereby regulating Keap1/Nrf2 pathway. This study helps to deepen our understanding of the role and function of m^6^A modification in colistin-induced nephrotoxicity, and provides a new idea for alleviating nephrotoxicity of colistin.

## Data Availability

The raw data supporting the conclusions of this manuscript will be made available by the authors, without undue reservation, to any qualified researcher.

## Author Contributions

JL and CC supervised the whole experiments. JW designed this study. JW, MI, and LX performed the practical work and completed the experiments. CX provided help during experiments. MI helped in revising and improving the language expression.

## Conflict of Interest Statement

The authors declare that the research was conducted in the absence of any commercial or financial relationships that could be construed as a potential conflict of interest.
