# Supplementary material for: METTL3/m6A/miRNA-873-5p Attenuated Oxidative Stress and Apoptosis in Colistin-Induced Kidney Injury by Modulating Keap1/Nrf2 Pathway
Source: Front Pharmacol. 2019 May 15;10:517. doi: 10.3389/fphar.2019.00517 (PMC6530351; doi:10.3389/fphar.2019.00517)
Supplement: Supplementary file 1 [file Data_Sheet_1.docx]

Supplementary Material

## Supplementary Figures


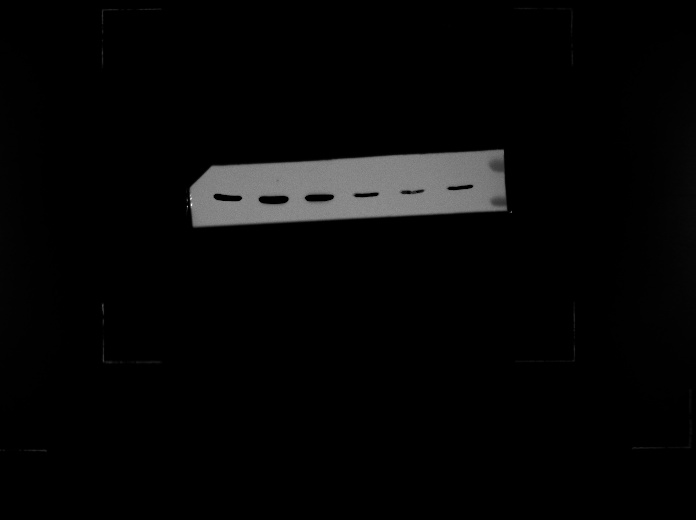


70KDa

55KDa

**Supplementary Figure 1.** Keap1 in Figure3.


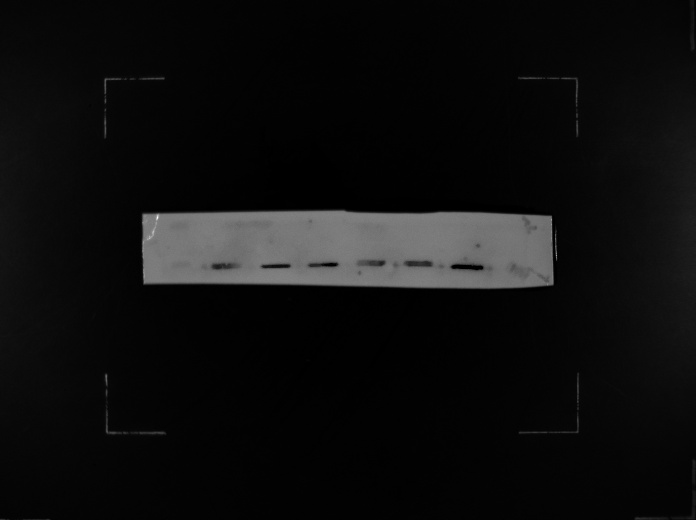


130KDa

170KDa

**Supplementary Figure 2.** Nrf2 in Figure3.


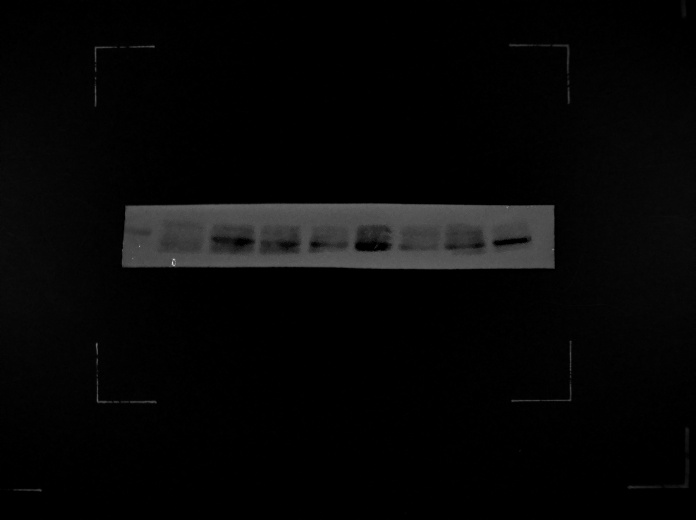


25KDa

35KDa

**Supplementary Figure 3.** HO-1 in Figure3.


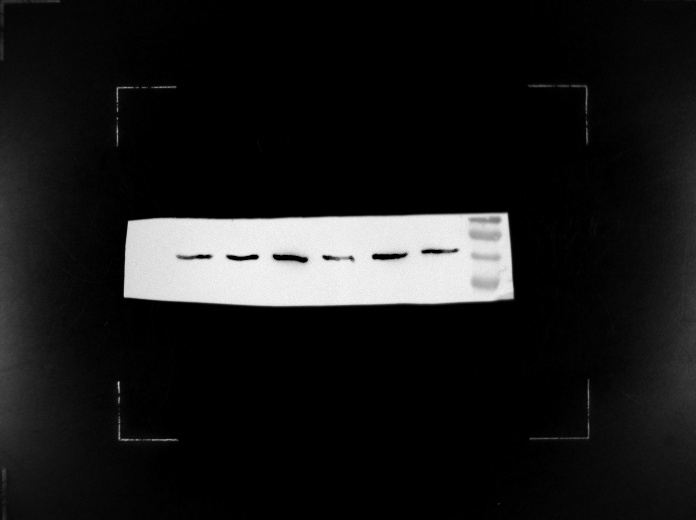


40KDa

55KDa

70KDa

100KDa

**Supplementary Figure 4.** Keap1 in Figure4.


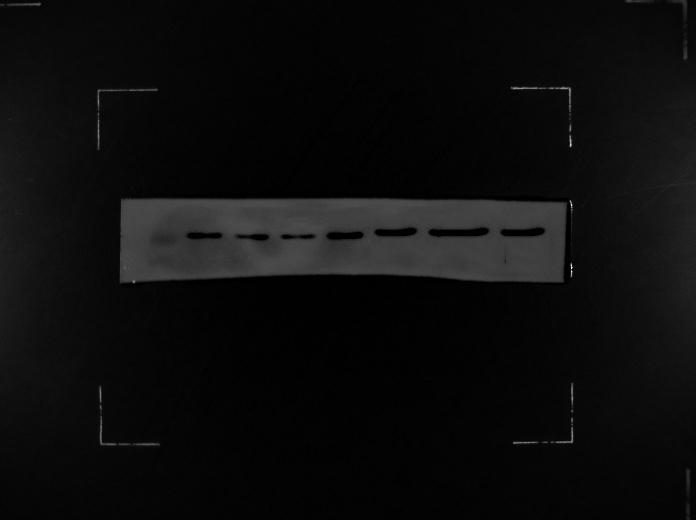


130KDa

**Supplementary Figure 5.** Nrf2 in Figure4.


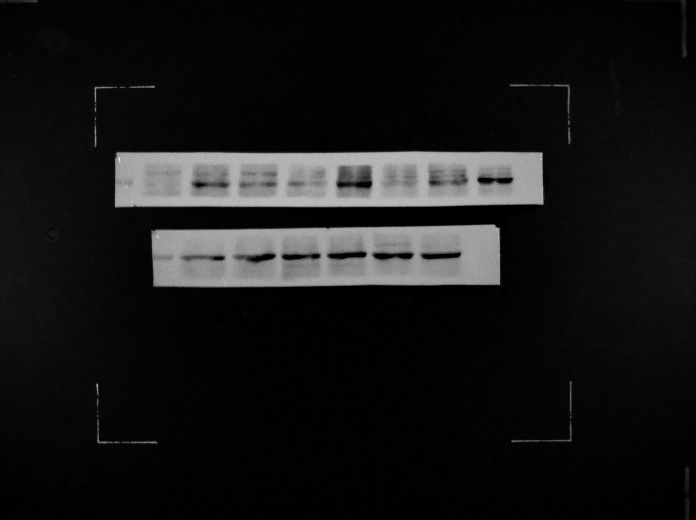


35KDa

**Supplementary Figure 6.** HO-1 in Figure4.


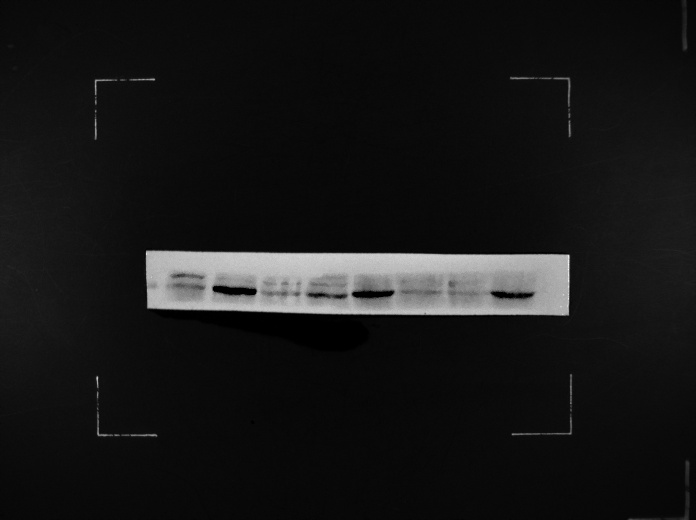


100KDa

70KDa

**Supplementary Figure 7.** DGCR8 in Figure4.


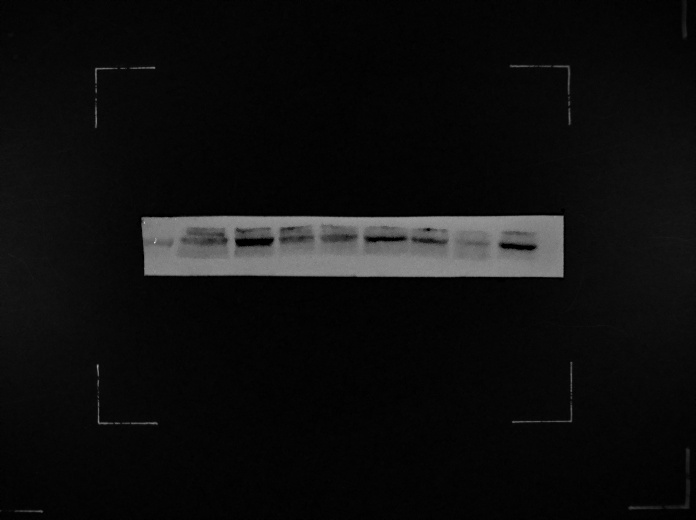


70KDa

**Supplementary Figure 8.** METTL3 in Figure 2 (red frame)and Figure 4 (white frame).


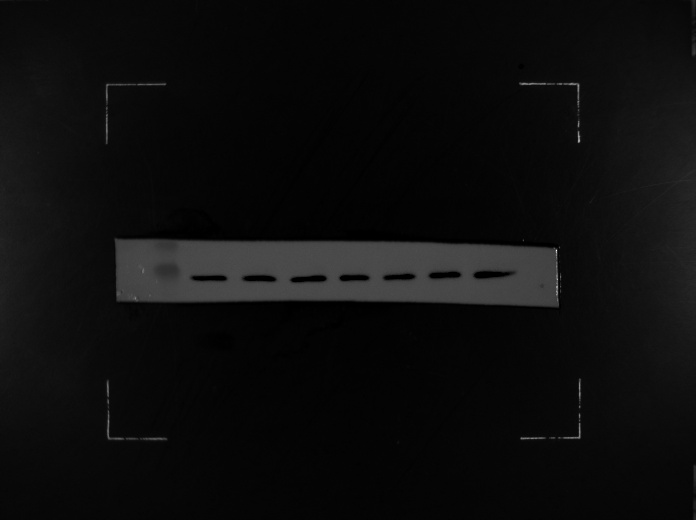


35KDa

40KDa

55KDa

**Supplementary Figure 9.** actin in Figure 2.


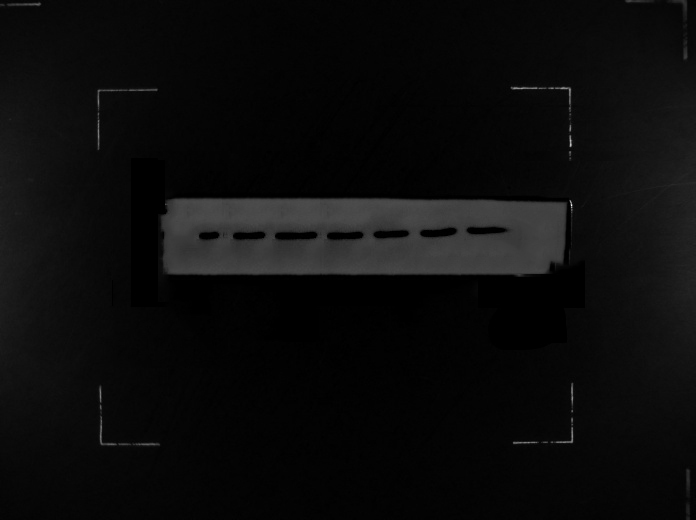


40KDa

**Supplementary Figure 10.** actin in Figure 3.


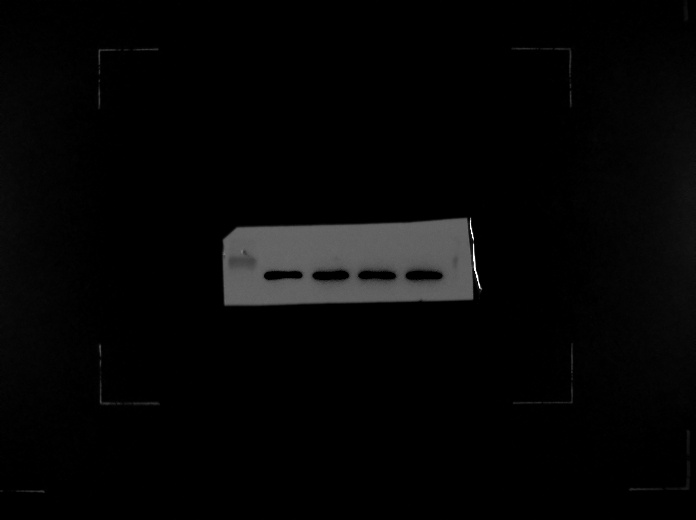


40KDa

**Supplementary Figure 11.** actin in Figure 4.
